# Population Dynamics of Cyanomyovirus in a Tropical Eutrophic Reservoir

**DOI:** 10.1264/jsme2.ME14039

**Published:** 2014-12-27

**Authors:** Bee Hui Yeo, Karina Yew-Hoong Gin

**Affiliations:** 1 School of Civil and Environmental EngineeringNanyang Technological UniversitySingapore; 2 Department of Civil and Environmental EngineeringNational University of SingaporeSingapore

**Keywords:** cyanophages, reservoir, cyanobacteria, phylogenetic diversity

## Abstract

Samples from three stations in Kranji Reservoir, Singapore (*n* = 21) were collected and analyzed for cyanomyovirus abundance and diversity. A total of 73 different g20 (viral capsid assembly protein genes) amino acid sequences were obtained from this study. A phylogenetic analysis revealed that the 73 segments were distributed in six major clusters (α to ζ), with four unique subclusters, which were identified as KRM-I, KRM-II, KRM-III, and KRM-IV. The cyanophage community in Kranji Reservoir exhibited a large degree of diversity; the clones obtained in this study showed similarities to those from many different environments, including oceans, lakes, bays, and paddy floodwater, as well as clones from paddy field soils. However, the sequences in this study were generally found to be more closely related to the g20 sequences of freshwaters and brackish waters than those from marine environments. The rarefaction curves and Chao 1 indices from this study showed that the diversity of the cyanomyovirus community was greater during the Inter-monsoon periods than the Southwest and Northeast Monsoons. A few seasonal changes in the taxa were observed: (i) Cluster ζ was absent during the Southwest Monsoon, and (ii) most of the samples fell into Group 3 in the PCA score plot during the Northeast Monsoon, and the fraction of Cluster ɛ increased significantly.

The discovery of an abundance of viruses in aquatic systems since the 1990s has re-initiated investigations into the ecological roles of viruses (6, 35). This re-initiated research has changed traditional and conceptual understanding of the function and regulation of aquatic ecosystems (15) with the realization of viruses as important players in aquatic ecosystems. Culture-independent approaches and molecular techniques have been chosen to better understand the diversity and distribution of viruses in the aquatic environment. Culture-based approaches require the maintenance and growth of a host organism (22) for viruses to infect. However, 95% of the bacteria reported to date cannot be cultivated (4). In contrast, molecular techniques allow researchers to directly characterize complex viral consortia (22).

The gene encoding the vertex portal protein (g20) in the *Myoviridae* family (the term cyanomyoviruses will be used hereafter for the sake of convenience) has been exploited to examine the diversity of natural cyanophage communities (14). Genetically similar g20 clones have been detected at distant sites with different nutrient status, temperatures, and salinities (11, 21). For example, clones from the Gulf of Mexico (marine environment, depth of 110 m) share 99% identity with g20 clones from Lake Constance (freshwater environment, depth of 3 m) (21). These findings suggest that some g20 clones are widely distributed with no apparent geographical segregation (11) or that horizontal gene exchanges occur among cyanophage communities (21). However, Sullivan *et al.* (23) suggested that g20 clones were distributed according to geographic segregation. For example, the population structure in an estuary and open ocean differed from each other and clonal diversity changed from the surface to the deep chlorophyll maximum (DCM) layer, as shown by Zhong *et al.* (39). Thus, similar g20 clones can be obtained from various environments, but g20 diversity distribution patterns (such as phylogenetic trees) from different environments differ and unique clades can be obtained from individual locations.

The temporal variation of cyanomyoviruses has been demonstrated from various environments *e.g.* Lake Bourget, Chesapeake Bay, the Red Sea, Norwegian coastal water, paddy flood water, and paddy field soils (5, 16, 19, 31–33). Mühling *et al.* (16) demonstrated that cyanomyovirus population diversity was greater during the spring and winter than the summer and autumn, and co-varied with *Synechococcus* diversity. Thus, previous studies showed both spatial and temporal variations in g20 clone distribution.

Singapore is a country with a tropical rainforest climate that is divided into four periods according to the average prevailing wind direction: Northeast Monsoon (NE) (December to early March), Inter Monsoon (late March to May), Southwest Monsoon (SW) (June to September), and second Inter Monsoon (October to November). Rainfall in Singapore generally begins to increase in October (monthly mean: 158.8 mm) and peaks in December (monthly mean: 329.5 mm) during the Northeast Monsoon (17). Kranji Reservoir is part of the Kranji catchment, which consists of different land-uses including residential areas, agriculture, forests, and reserve areas (10). Chua *et al.* (3) demonstrated that surface runoff loadings from the Kranji Catchment positively correlated with peak flow. Gin *et al.* (9) also suggested that heavier precipitation during the Northeast Monsoon affected the environmental parameters by dilution. Therefore, reservoir waters in Singapore face temporal changes due to the monsoon seasons. Kranji Reservoir has been categorized as a lake between a eutrophic and hypereutrophic status (26). Diverse cyanobacteria populations have previously been detected (Aug 2008 to Feb 2009), and species included *Microcystis*, *Anabaena*, and *Synechococcus* (27).

The objective of this study was to obtain a clearer understanding of the diversity and dynamics of cyanomyoviruses in Kranji Reservoir. Cyanomyovirus diversity was investigated using a PCR-cloning-sequencing approach with PCR primers targeting the g20 gene, which is myoviral-specific. In this study, we reported, for the first time, the distribution of g20 clones in tropical reservoir water. We also investigate the effects of monsoon seasons on g20 clone distribution and determined differences in the genetic makeup of cyanomyovirus populations in a Singapore reservoir and other aquatic environments.

## Materials and Methods

### Sampling site and field sampling

**Fig. 1:**
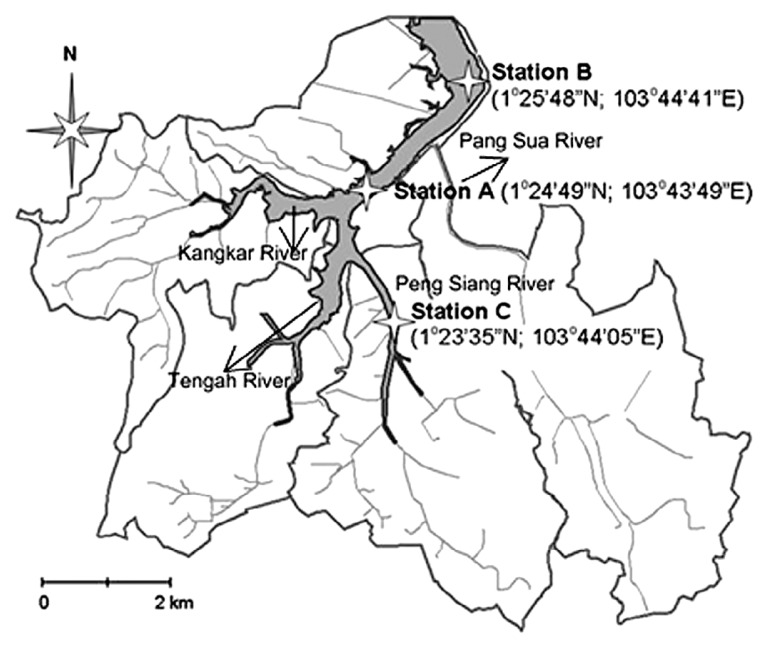
Map of sampling locations (Station A, Station B, and Station C) in Kranji Reservoir, Singapore.

Kranji Reservoir was formed by damming the mouth of the Kranji estuary. The reservoir has a water surface area of approximately 2.8 km^2^ and is mainly supplied by three tributaries in the southern part of the reservoir: Tengah River, Kangkar River, and Peng Siang River. Water flow from Pang Sua River was diverted into Kranji Reservoir at the end of 2005 as the fourth tributary at the southern end of the reservoir (3). Three sampling stations were chosen, *i.e.* Station A (1°24′49″N; 103°43′49″E), Station B (1°25′48″N; 103°44′41″E), and Station C (1°23′35″N; 103°44′05″E) (Fig. 1). These three sampling locations represent different hydrological conditions. Station A is located at the confluence of the three tributaries (excluding Pang Sua River); Station B is located at the end of the reservoir, close to the Straits of Johor and Kranji Reservoir Park; and Station C is located in the middle of the Peng Siang tributary. These three stations also have different depths, with the maximum depth of Station C being only one meter deep while the deepest point is located at Station B at approximately 14 m.

Global Positioning System (GPS) was used to locate the selected sites. Approximately 12 L of surface water was collected monthly from Kranji Reservoir between August 2008 and February 2009 to investigate the abundance and population dynamics of cyanophage communities. Another 1 L of surface water from Stations A, B, and C was collected for the purpose of water quality parameter measurements, kept in an ice box (~4°C), and transported to the laboratory. *In situ* measurements were also conducted and recorded on site: water temperature, dissolved oxygen (DO), salinity, conductivity, Secchi depth, and total dissolved solid (TDS). A YSI meter (EC300) was used to measure water temperature, salinity, and TDS and a YSI model 52 (YSI probe 5739) was used to measure the dissolved oxygen concentration. Secchi depth or water transparency was determined with a Secchi disc.

### Concentration of viral communities and DNA extraction

A total of 12 L of surface water was collected with autoclaved, sample-rinsed carboys and transported to the laboratory immediately. Briefly, 12 L of sample was gently filtered through a 50 or 20-μm pore size with low binding protein membrane filters (Pall Corporation) to remove particulate matter, phytoplankton, and zooplankton. The filtrate was subsequently filtered through a 0.2-μm pore size membrane (Pall Corporation) to remove most of the bacteria. After undergoing pre-filtration, samples were stored in a 4°C cool room. The viruses in the filtrate of approximately 10 L of sample were concentrated to a final volume ranging from 250 to 500 mL using tangential flow filtration (TFF) with a 30 kDa membrane cassette (Pall Corporation). Secondary concentration was performed using an Amicon Ultra centrifugal filter device (Millipore). Briefly, the TFF-concentrated sample was loaded to the filter device (10 kDa) and centrifuged at 5,000 × *g* for approximately 10 min according to the manufacturer’s instructions. The final volume of each concentrated water sample was approximately 4 mL from 200 mL for DNA extraction purposes. Concentrated samples after the second concentration were stored in the dark at 4°C until used. These samples were believed to contain virus particles and ready for DNA extraction.

Viral DNA was extracted from 200 μL of the concentrated sample using the DNA extraction kit (Qiagen, QIAamp DNA Mini Kit) according to the manufacturer’s instructions. Viral DNA was eluted in 60 μL of DNase- and RNase-free buffer and stored at −20°C until the molecular work was performed. The extracted DNA purification efficiency *i.e.* DNA concentration and purities were measured with a spectrophotometer (NanoDrop, ND1000 from NanoDrop Technologies) at an absorbance of 260 nm and ratio 260 nm/280 nm (A260/A280), respectively.

### Environmental parameters

Water quality parameters were measured at each station and date according to standard methods, *i.e.* American Public Health Association (2) and United States Environmental Protection Agency (29). The water quality parameters examined included total nitrogen (TN), total phosphorus (TP), calcium ions (Ca^2+^), magnesium ions (Mg^2+^), turbidity, total suspended solid (TSS), pH, and chlorophyll-a (Chla). Chla was measured according to APHA 10200H (1) with modifications. In brief, 50–250 mL of the water sample with MgCO_3_ (0.001% w/v) added was filtered through a cellulose nitrate membrane. Subsequently, 10 mL of 90% acetone was added to the membrane, then sonicated for 3 min and incubated in the dark for 24 h. After 24 h, the sample was then centrifuged at 2800 rpm for 15 min. A clear supernatant (chlorophyll-*a* extracted by acetone) was obtained and measured with a spectrophotometer (Beckman Coulter DU 640B).

### Polymerase chain reaction (PCR)

PCR amplification was performed with CPS1/CPS8 primers (39) to detect the g20 gene. Amplification reactions were carried out in 50-μL volumes containing 1x Taq polymerase buffer (Promega), 4 mM MgCl_2_ , 0.2 mM of a deoxynucleosidetriphosphate (dNTP) mix, 0.5 μM of each primer, 2.5 units Taq polymerase with hot start (Promega), and 5 μL template DNA. PCR amplification was carried out with a Mastercycler®pro thermal cycler from Eppendorf and performed with the following cycle profile: initial denaturation at 94°C for 3 min, 36 cycles of denaturation at 94°C for 45 s, annealing at 36°C for 45 s, extension at 72°C for 1 min, and a final extension at 72°C for 5 min.

Final PCR products were run on a 1.5% agarose gel in 1x Tris-Borate-EDTA stained with ethidium bromide for 1 h at 90 volts. The gel was visualized under UV trans-illumination and photographed to confirm the target amplicon. The correct base pair for the CPS1/CPS8 amplicon was *ca.* 592 bp.

### Quantitative real-time PCR (qPCR)

A pair of g20 gene primers (CPS1 and CPS2) designed by Fuller *et al.* (7) specifically amplifies a gene encoding a capsid assembly protein (g20) belonging to cyanophages in the *Myoviridae* family. Matteson *et al.* (13) recently verified the specificity of CPS1/CPS2 primers (g20 primers used in this qPCR study) using DE-METAST-BLAST. Their findings confirmed that these primers could only amplify cyanomyovirus amplicons based on sequences in the Global Ocean Survey data set.

The standard used to determine the gene copy number of the g20 gene was based on the cloned plasmids using the specific sequence for g20, and the standard curve was obtained from the calculated plasmid concentration at various dilutions. The quantification of cyanomyoviruses that encode the g20 gene (CPS1/CPS2) was performed with 5 μL of DNA template, 0.5 μM of both primers, 10 μL of 2x reaction master mix (FastStart Universal SYBR Green Master Mix [ROX], Roche), and sterilized distilled water to a final volume of 20 μL. The thermal cycle and measurement of SYBR Green fluorescence binding were performed in a Light-Cycler qPCR using the following program: pre-incubation at 95°C for 15 min; amplification for 45 cycles consisting of denaturing at 95°C for 15 s, annealing at 58°C for 20 s, and extension at 72°C for 30 s; melting curve analysis (1 cycle amplification) heated to 95°C, annealing at 65°C for 60 s, and extension at 95°C. Only 6 samples from each station (total *n=*18) were enumerated for g20 gene concentration, *i.e.* with the exception of the December 2008 sample (this was due to a change in the qPCR machine leading to inconsistent results).

The qPCR products were run on a 3% agarose gel in 1x Tris-borate-EDTA (TBE buffer) stained with ethidium bromide at 100 volts for 1 h. The gels were photographed under UV transillumination with a camera (Kodak DC 290). The correct g20 qPCR amplicon was placed at *ca.* 165 bp.

### Cloning

A pair of family-specific gene primers (CPS1 and CPS8) was used in this study. These primers were developed by Zhong *et al.* (39). These primers have been used to study the diversity of cyanomyoviruses infectious to *Synechococcus* and *Prochlorococcus* (23). However, not all cyanomyoviruses infectious to *Synechococcus* and *Prochlorococcus* were able to be detected by these primers (23).

The PCR amplicons from CPS1/CPS8 amplification were purified using the Wizard® SV Gel and PCR clean up system (Promega, Corporation, Madison, WI). Purified PCR products were subsequently cloned into the pGEM-T Vector System II (Promega) and the construct was transformed into JM109 competent cells according to the manufacturer’s instructions. At least eight clones were randomly picked up from each sample and the correct target fragments were confirmed using PCR. At least four successful transformants were magnified overnight in LB broth with ampicillin. Plasmid DNA was extracted from the competent cells using the Wizard® SV Minipreps DNA Purification System (Promega). The extracted plasmid DNA was then sent for sequencing. All sequencing services were provided by AITbiotech Company.

### Phylogenetic analysis

All nucleotide sequences were edited and aligned with the BioEdit sequence alignment editor (http://www.mbio.ncsu.edu/bioedit/bioedit.html). After editing, sequences were compared with cyanophage prototypes and to other reference sequences available in the GenBank database using BLAST software. Subsequently, all sequences from this study together with some previously obtained cyanomyovirus isolate g20 gene sequences and uncultured g20 clone sequences from other environments were aligned with ClustalW Multiple Alignment and saved in the FAS format. A phylogenetic tree was constructed with MEGA 5 (25) using the aligned sequences. The phylogenetic tree was drawn using the neighbor-joining method with *p*-distance and 1,000 bootstrap replications.

The Chao-1 index and rarefaction curve were obtained for monsoons using PAST software (http://folk.uio.no/ohammer/past).

### Statistical analysis

A Principal Component Analysis (PCA) was carried out using the PASW 18 software package (SPSS). The PCA analysis was used to compare samples collected from different sampling locations and different sampling periods. Six environmental variables (*i.e.* TN, TP, Chl, Turbidity, Secchi depth, and TSS) were chosen in this PCA analysis. These variables were previously reported to be significantly affected by cyanobacteria concentrations in Kranji Reservoir (27).

### Nucleotide sequence accession numbers

The g20 sequences from this study were deposited in the GenBank database. The GenBank accession numbers for the sequences are KC485882–KC485966.

## Results

### Environmental characteristics

Tables S1 to S3 show the environmental variables monitored during this study. Table 1 shows the summary of environmental variables at Kranji Reservoir. Significantly higher concentrations of total nitrogen (TN) and total phosphorus (TP) were observed at Station C than at Stations A and B. The concentration of chlorophyll-a markedly increased at Station C between October 2008 (111 μg L^−1^) and November 2008 (1,585 μg L^−1^), and then remained between 76 μg L^−1^ and 184 μg L^−1^ (from December 2008 to February 2009).

**Fig. 2:**
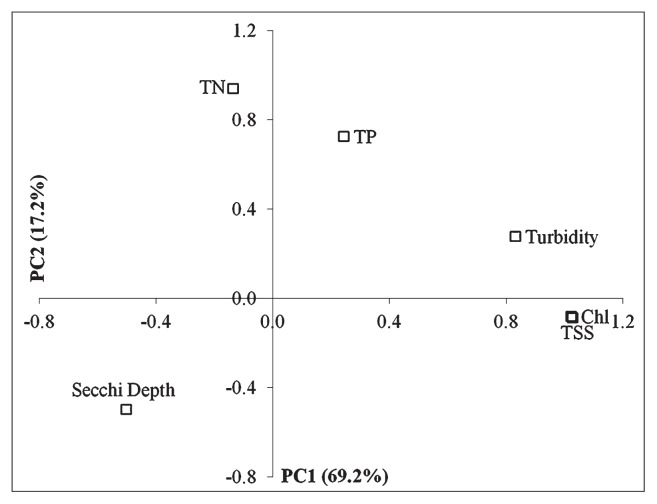
PCA ordination plot of variables for chlorophyll-a (Chl), total suspended solid (TSS), turbidity, Secchi depth, total nitrogen (TN), and total phosphorus (TP). The first component (PC1) explained 69.2% of the data variance and was mainly defined by Chl-a, TSS, turbidity, and Secchi depth. The second component (PC2) was mainly defined by TN and TP and explained 17.2% of the data variance.

PCA was performed using 6 variables based on 21 complete data sets. Kaiser-Meyer-Olkin Measure of Sampling Adequacy (KMO) together with Bartlett’s test of Sphericity were used to check the suitability of the analysis. The value of KMO was 0.728 (> 0.6) and Bartlett’s Test of Sphericity was significant (*P* < 0.0005), showing that the analysis was appropriate (18). Fig. 2 shows the PCA ordination plot on which the first component (PC1=69.2%) and second component (PC2 = 17.2%) accounted for 86.4% of the data variation. PC1 was mainly defined by phytoplankton biomass-related variables including chlorophyll-a, TSS, turbidity, and Secchi depth. PC2 was mainly influenced by nutrient (TN and TP).

### Cyanomyovirus abundance

**Fig. 3:**
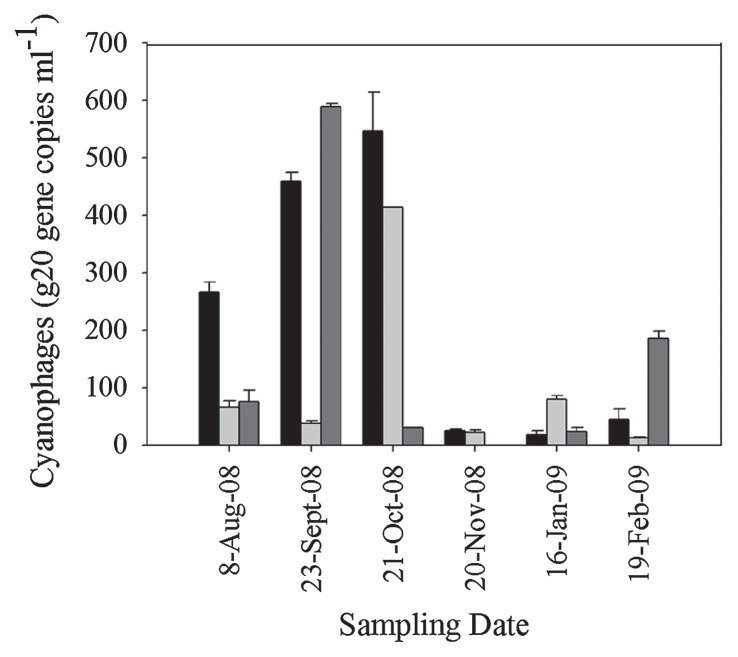
Dynamics of cyanomyoviruses (estimated by qPCR using CPS1/CPS2 primers) at Station A (bars in black), Station B (bars in light grey), and Station C (bars in grey) from August 2008 to February 2009. Error bars represent the standard deviation of triplicate data from the qPCR analysis.

We quantified cyanomyoviruses using CPS1/2 primers. The estimation of putative cyanomyovirus abundance based on the g20 gene showed that the range varied temporally and between stations from below the detection limit to 6 × 10^2^ gene copies mL^−1^ (Fig. 3). Only the sample in November 2008 from Station C was below the detection limit, but showed the presence of the g20 gene. A total of six sampling sites from four previous studies enumerated the g20 gene by employing the primer set CPS1/2, *i.e.* a Norwegian coast (19), Lake Erie (Laurentian Great Lake, North America) (12), Lake Annecy, Lake Bourget (38), and the Sargasso Sea and Southern Pacific Ocean (13). Our study showed that the g20 gene density in Kranji Reservoir was closer to Norwegian coastal water (5.0 × 10 to 7.2 × 10^3^ gene copies mL^−1^) and lower than the other sampling sites, ranging from 10^2^ to 10^5^ gene copies mL^−1^.

### Analysis of nucleotide and amino acid g20 sequences

A total of 88 g20 clones were sequenced in this study and 86 clones were sequenced as g20 fragments with 33 clones from Station A, 27 clones from Station B, and 26 clones from Station C. The remaining two sequences showed 80% similarity to environmental clones, but both of these sequences encoded a stop codon, which was absent in the other sequences. Therefore, these two clones were removed from the phylogenetic analysis and were not considered as g20 fragments. Therefore, g20 recovery efficiency was 97.7% (86/88).

The nucleotide sequence analysis revealed seven pairs of identical sequences and one set with three identical sequences at the nucleotide level (100%). All pairs of identical nucleotide sequences originated from the same sample, such as KRA1008M1 and KRA1008M2, which were 100% identical in their sequences and were from Station A on 21 October 2008. The 3 clones with identical nucleotide sequences were obtained on the same sampling date (16 January 2009), but from three different locations. The amino acid sequence analysis revealed five pairs of identical amino acid sequences. Only one of the five pairs was from the same sample, while the rest were from different sampling dates, locations, or both.

Sequence analyses of a total of 86 different g20 segments revealed that within the examined gene fragment, the most variable region consisted of an insertion/deletion site, which varied between sequences by up to four amino acids. The lengths of the g20 fragment varied among clones (between the primers): 546 bp for 64 clones (74.5%), 549 bp for 7 clones (8%), 552 bp for 14 clones (16.3%), and 554 bp for 1 clone. The identity within the clones in this study at the amino acid level ranged from 50% to 99%. The normal g20 fragment length was 546 bp. G20 fragments with 549 bp have also been identified from g20 clones from paddy floodwater and paddy field soils (31, 32), while those with 552 bp were reported only from paddy soil samples.

The BLAST search within NCBI showed that all our sequences at the amino acid level were under a putative conserved domain called Phage_T4_Gp20_Superfamily. Table S4 shows a summary of the closest relatives at the amino acid level, revealing that this study had the highest identities, varying from 60% to 96% with the g20 clones obtained from other environments (as listed in Table S4). The clone names were labeled based on their location, sampling date, family (*Myoviridae*), and sample number. For example, KRA0808M1 indicated clone sample 1 of the g20 fragment (M-*Myoviridae*) from Station A collected on August 2008. KRB and KRC represented Stations B and C respectively.

Table S4 shows that the clones from our study mostly had the highest similarities with the clones from fresh waters (*e.g.*, Dianchi Lake, Cultus Lake, Lake Bourget, paddy field floodwater, and paddy field soil) and brackish waters (*e.g.*, Sandusky Bay, Cheasapeake Bay, Skidaway Estuarine, and ballast water). Only four clones (< 5%) from this study had the highest similarities with marine environments (*i.e.*, Atlantic Meridional Transect, DCM of Sargasso Sea and Beaufort Sea, Arctic Ocean). The highest identity clones compared favorably with the study by Wilhelm *et al.*, (36), especially clones from Station B: 65.4% of the Station B clones had the highest similarities with the ballast water and Sandusky Bay clones obtained by Wilhelm *et al.* (2006). There were only two clones from Station B that showed the highest similarities with paddy field clones. However, Station A had 13 clones (48%) and Station C had 10 clones (38%) with the highest similarities with paddy field clones.

### Phylogenetic analyses

**Fig. 4:**
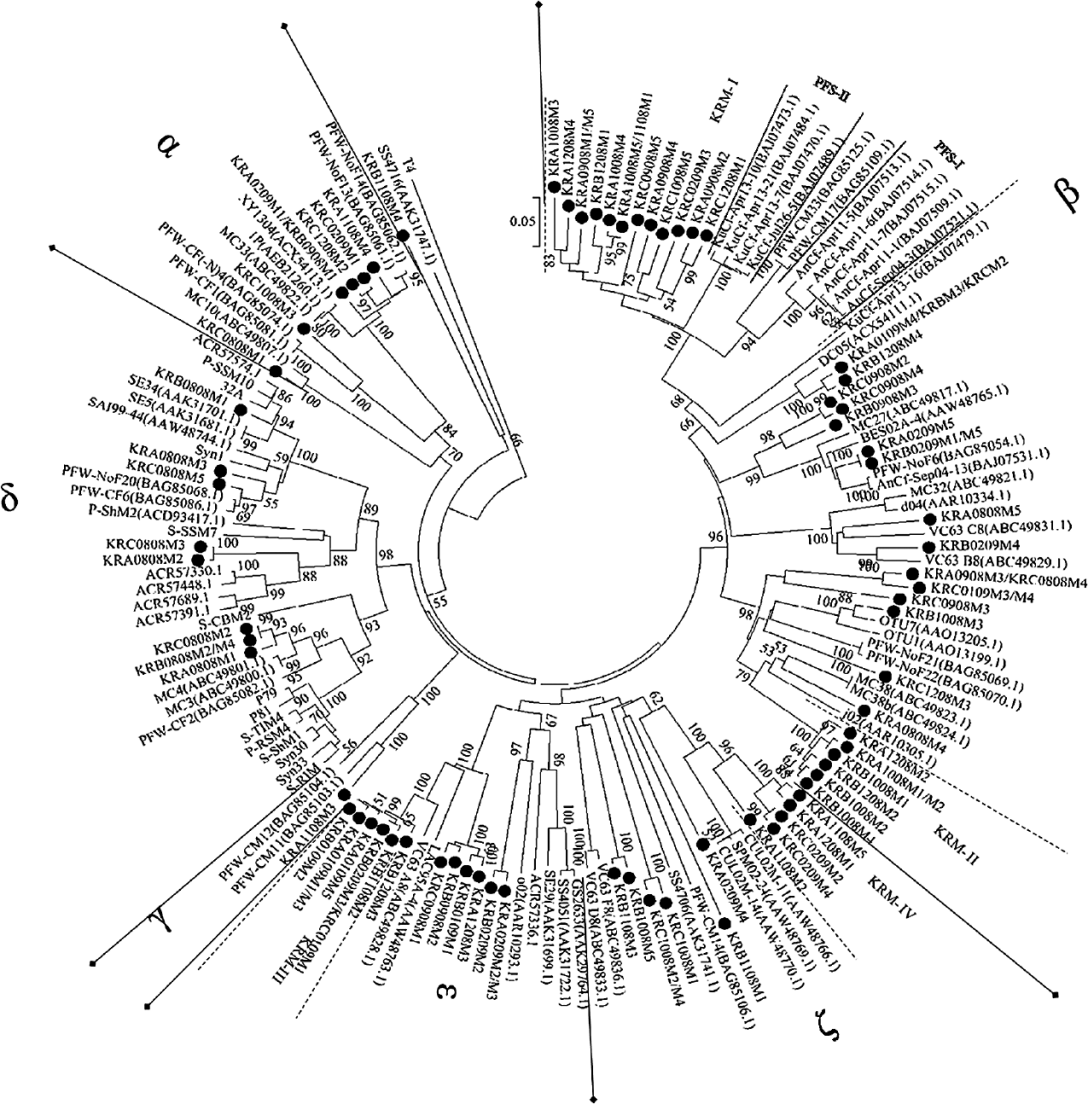
Neighbor-joining phylogenetic tree of the g20 gene amino acid sequence showing relationships between g20 amino acid sequences from Kranji Reservoir and other environmental sequences. Black circles indicate clones obtained in this study. Numbers in the parentheses are the accession numbers of amino acid sequences in the NCBI web site. The percentage of replicate trees in which the associated taxa clustered together in the bootstrap test (1000 replicates) are shown next to the branches (25). Bootstrap numbers less than 50 are not shown in the phylogenetic tree. The clone names are labeled according to the format WXYZ, where W is the sampling location, X is the sampling date, Y is the phage family (*Myoviridae*), and Z is the sample number.

In this study, after merging clones with identical amino acid sequences, 73 different g20 segments were obtained and used for phylogenetic analyses. Phylogenetic analyses were carried out based on alignments consisting of these 73 Kranji Reservoir g20 segments and 80 sequences from previous studies. The addition of the g20 segments from other environmental studies was to ensure that the representative sequences included most of the g20 phylogenetic lineage identified to date. The phylogenetic tree (Fig. 4) revealed that the g20 clones obtained from Kranji Reservoir were distributed into six major clusters (α to ζ). This assignment corresponded to previously reported g20 clones from marine water, freshwater, ballast water, paddy floodwater, and paddy field soil environments. The phylogenetic tree clustering of the g20 clones was consistent with previous findings, except that Cluster ζ was newly assigned. Clusters α to ɛ were clustered according to Wang *et al.* (31). One sequence, KRB1108M4, stood apart from these six clusters.

The following observations were made with reference to the phylogenetic tree (Fig. 4). Cluster α, with a bootstrap of 70%, contained clones from lakes (30, 37), paddy floodwater (31), Sandusky Bay (36), and this study (7 out of 86 clones). Cluster α exclusively included clones from Kranji Reservoir and clones from freshwater environments only. This was supported by Wang *et al.* (31), who also found that cluster α was specific to g20 genes from freshwater environments including Sandusky Bay particulates and floodwater from paddy fields.

Cluster β was the largest cluster and strongly supported (96%). It was observed for all sampling dates and, hence, could be the dominant species in Kranji Reservoir. It contained clones from freshwater environments (5, 31, 32, 36, 37), brackish waters (33), marine environments (21), and this study (41 clones, 48%). In this study, two unique subclusters were identified within Cluster β: KRM-I and KRM-II. Kranji Reservoir clones (14 clones, 16.5%) formed a weakly supporting (47%) independent sister clade in subcluster KRM-I. This clade with another two sister clades as assigned by Wang *et al.* (32), PFS-I and PFS-II, established the strong bootstrap (100%) subcluster KRM-I in Cluster β. KRM-II was a strongly supported (100%) subcluster that contained seven clones from this study.

Cluster δ, also known as cluster CSP, was first designated by Short and Suttle (21). Cluster CSP contained g20 sequences from cyanophage isolates which infect marine *Synechococcus* and *Prochlorococcus* (21, 31). Cluster δ in this phylogenetic tree consisted of clones from marine waters (11, 23, 24, 39), freshwaters (36), and paddy floodwaters (31). This cluster was strongly supported (99%) and contained nine clones (11%) obtained from this study (all clones from August 2008 samples).

Cluster ϒ was the smallest cluster, but with strong bootstrap support (100%). Cluster ϒ only contained a clone from this study and two clones (PFW-CM11 and PFW-CM12) from paddy floodwaters (31). PFW-CM11 and PFW-CM12 formed a unique subcluster, PFW-IV, in Wang *et al.* (31)’s study.

Cluster ɛ was a weakly bootstrap-supported (40%) cluster that accounted for 25% of the 85 clones from this study. Cluster ɛ consisted of clones from the deep chlorophyll maximum (DCM) of the Sargasso Sea, Gulf Stream (39), Atlantic Ocean (11), Sandusky Bay (36), and lakes (5). Another subcluster within Cluster ɛ, consisting only of Kranji Reservoir clones, has been identified and named as KRM-III. KRM-III was a strongly supported (99%) subcluster consisting of eight clones (9.4%) from this study.

Cluster ζ was a newly identified cluster in this study. Cluster ζ with very weak bootstrap support (15%) contained clones from the DCM of the Sargasso Sea (39), Arctic cyanobacterial mat (21), paddy floodwater (31), paddy field soils (32), bay water (36), lake water (21), and clones from this study (12 clones, 14%). A subcluster within Cluster ζ consisting only of Kranji Reservoir clones was identified and named KRM-IV. KRM-IV was a strongly supported (96%) subcluster.

Clone KRB1108M4 was unique and not grouped to any cluster. It was most closely related to the DCM clone from the Sargasso Sea clone (SS4716).

### Spatial changes in the cyanomyovirus community

In this study, the cluster distribution of cyanomyovirus communities was shown across the three locations (Station A, Station B, and Station C) based on the clones produced. The spatial cluster distribution in this study (Table 2) did not reveal differences in the overall patterns of the composition of cyanomyoviral assemblages for these three stations in Kranji Reservoir.

However, a certain degree of variation was observed in the proportion of clones for each cluster: (1) the proportion of cyanomyoviruses that belonged to Cluster ɛ were higher at Station B (7 out of 15 clones), (2) Station C had the highest proportion of Cluster α clones (4 out of 7), and (3) Station C had the lowest proportion of Cluster ɛ clones (2 out of 15).

### Temporal changes in the cyanomyovirus community

The temporal cluster distribution of the myoviral cyanophage population is shown in Table 3. Between three to four g20 clusters were detected in samples collected from August 2008 to February 2009, except for November 2008 and January 2009.

The November 2008 sample from Station C was the only sample that showed no amplification when CPS1/CPS8 primers were used in PCR, and the sample concentration was under the detection limit when CPS1/CPS2 primers were used in qPCR. At this particular sampling site and date, the cyanobacterial bloom (with obvious scum) had the highest chlorophyll-a concentration (1,584 μg L^−1^) and was an extreme case in the PCA. The November 2008 samples (from Stations A and B) exhibited greater genetic diversity than the other 6 months. Only 9 clones were obtained from the November 2008 samples, but these clones were distributed to 5 different clusters and six genotypes, and KRB1108M4 was absent in all clusters. In contrast, only two cyanophage genotypes (clusters β and ɛ) were detected in January 2009 when 11 clones had been sequenced.

The sequences belonging to Cluster δ only occurred in August 2008 and only one clone was obtained in November 2008 belonging to cluster ϒ. Cluster α was absent in January 2009 while cluster ɛ was absent in August 2008. The cluster α genotype was present for six months, but only consisted of seven clones, except for February 2009, during which two clones were detected. Cluster β was the only genotype present for all sampling dates; however, spatially, Cluster β was absent at Station B in August and November 2008. Cluster β was the dominant fraction in the September, October, and December 2008 and February 2009 clones.

**Fig. 5:**
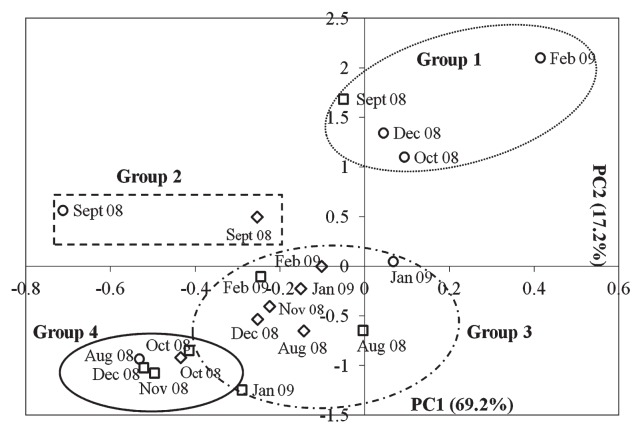
Factor analysis scores for the PCA plot. Station C (November 2008) (with score PC1:4.21467; PC2: 1.27372) was not shown in the plot as it was an anomaly resulting in the clustering of the plots, making them difficult to differentiate. Station A (open diamond), Station B (open square), and Station C (open circle) are grouped into four groups. Group 1 and Group 2 consist of samples more closely related to g20 clones from paddy floodwater and paddy field soil. Group 3 consists of samples with the g20 sequences more closely related to g20 clones from lakes and bays. The g20 sequences in Group 4 samples had the most diverse clones, with these clones being closely related to different environments, including clones from bays, paddy field soil, ballast water, paddy floodwater, and marine water.

The score plot of samples is shown in Fig. 5, in which samples were grouped into four groups. Groups 1 and 2 consisted of samples with high numbers of g20 sequences falling into cluster β and ζ (more closely related to g20 clones from paddy floodwater and paddy field soil). Group 3 consisted of samples with g20 sequences more closely related to g20 clones from lakes and bays. Group 3 samples were mainly from the NE monsoon. Group 4 samples had the most diverse clones, with clones closely related to different environments, including bays, paddy field soil, ballast water, paddy floodwater and marine water.

Table 4 shows the fraction and distribution of clusters for the three different monsoons, *i.e.* SW Monsoon, Inter Monsoon, and NE Monsoon. The SW Monsoon in this study was represented by six samples with a total of 26 clones. The Inter Monsoon consisted of six samples over two months, with 23 clones produced from 5 samples (note that the Station C November 2008 sample did not give any amplification). A total of 36 clones obtained from 9 samples over three months represented the NE Monsoon.

Cluster β was the major cluster for the three different monsoon seasons. Cluster ζ only appeared from October 2008 onwards, and was absent in the SW Monsoon Season (August and September 2008), in which a total of 26 clones was investigated. Cluster ζ was more dominant during the Inter Monsoon period than the NE Monsoon. The fraction of Cluster ɛ increased significantly in the NE Monsoon. Twelve clones from the NE Monsoon fell into this cluster, while only two clones and one clone came from the SW Monsoon and Inter Monsoon, respectively. Subcluster KRM-III in Cluster ɛ only consisted of clones from the NE Monsoon.

**Fig. 6:**
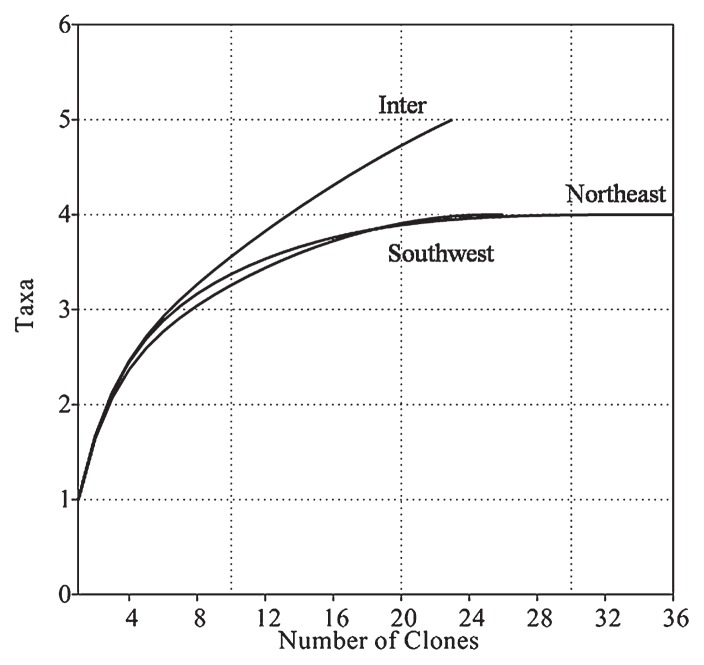
Rarefaction curves of cyanomyoviruses for different monsoon seasons.

Fig. 6 shows the rarefaction curves for three monsoon seasons. The SW and NE monsoon rarefaction curves reached an asymptote. However, the Inter Monsoon rarefaction curve did not reach an asymptote, thus suggesting that the diversity of g20 sequences was greater for the Intermonsoon. The rarefaction curve results were supported by the Chao 1 index. The Chao 1 index is a non-parametric richness estimator that can be used to determine total richness (20). Chao 1 indices (mean) for the SW, NE, and Inter-monsoons were 4, 4, and 5.5 respectively. Clones for the SW and NE Monsoons were distributed to four clusters (taxa), as shown in Table 4. The clones obtained from this study were distributed into five clusters for the Inter-monsoon, with a Chao 1 index of 5.5, suggesting that greater diversity was present during the Inter Monsoon.

## Discussion

Despite the important ecological roles played by cyanophages, data regarding the occurrence of cyanophages in tropical aquatic environments in Southeast Asian is lacking. The prevalence of cyanophages in a tropical eutrophic reservoir was examined in the present study. Local environmental g20 clones were obtained and compared with isolates from other environments using a phylogenetic analysis. Temporal and spatial changes in cluster distribution were obtained and analyzed according to changes in environmental parameters.

### Phylogenetic diversity of g20 gene sequences in Kranji Reservoir

The cyanophage community in Kranji Reservoir revealed a large degree of diversity. Clones from this study showed similarities to those from different environments, including oceans, lakes, bays, paddy floodwater, and paddy field soil. A previous study on phytoplankton structures in Singapore reported that different *Synechococcus* communities were detected in the Singapore Strait and Johor Strait (8), and the Johor Strait was more eutrophic and had higher nutrient levels than the Singapore Strait. Different *Synechococcus* strains (a total of 9 *Synechococcus* clones) were present in Kranji Reservoir, including *Synechococcus* belonging to clones collected from a hydroelectric power plant reservoir, lakes, reservoir, and Chesapeake Bay (27). The richness of the genotypes of cyanobacteria in Kranji Reservoir is suggested to contribute to the large degree of diversity in this tropical cyanophage community.

### Population dynamics of cyanomyoviruses

CPS1/CPS8 primers have successfully identified *Synechococcus* and *Prochlorococcus* phages by detecting the presence of the g20 gene (23, 39). However, no study to date has shown that these primers can detect *Microcystis* and *Anabaena* phages. The CPS1/CPS8 amplicons from this study are believed to originate from the g20 genes of cyanophages, which only infect *Prochlorococcus* or *Synechococcus*.

The temporal variations in cyanomyovirus cluster distribution exhibited significant temporal variations. Samples from August 2008 in cluster δ were closely related to known marine cyanomyovirus isolates. In this study, only 9 out of the 86 g20 clones examined belonged to cluster δ, indicating that marine type *Synechococcus* and *Prochlorococcus* were generally not predominant in Kranji Reservoir, except for August 2008. Table 3 shows that the Cluster β fraction markedly increased from September 2008, with the development of a unique sister clade in subcluster KRM-I (Fig. 4). Clones from this sister clade were more closely related to the clones in PFS-I. Wang *et al.* (32) previously suggested that Cluster β was the main cluster of the g20 gene from paddy field soils with PFS-I as a unique group. These changes in cluster distribution suggested that the *Synechococcus* community from Kranji Reservoir was more closely related to the *Synechococcus* community from paddy field soil. The PCA score plot (Fig. 5) also showed that the August 2008 and September 2008 samples were clustered into different groups. This temporal variation may shed some light on the role of cyanophages as indicators of shifts in the host community.

### Phages and host interaction

The concept of “killing the winner” was proposed by Thingstad and Lignell (28) through a theoretical model. “Killing the winner” is an expression used to describe the process by which lytic viruses lyse the most successful population (fastest growing population) and allow for the co-existence of less competitive populations, thereby sustaining bacterial diversity (13, 34). The g20 gene concentration for Stations A and B showed a maximum in October (Fig. 3) and the proportions of the dominant cluster were 100% and 80%, respectively (Table S4). In November 2008, Stations A and B showed a significant decrease in the abundance of g20 genes and the fraction of Cluster β. Nine clones from Stations A and B were simultaneously classified into five different clusters, revealing a more diverse cyanomyovirus community. The PCA score plot showed few differences in the water conditions in these two months.

The “killing the winner” phenomenon may have occurred between October and November at these locations. In October, phages present in Kranji Reservoir that were able to infect the vulnerable dominant host could replicate effectively at a high contact rate, resulting in high concentrations, but low numbers of cyanomyovirus genotypes. The dominant species was subsequently lysed and a new *Synechococcus* community was formed. Mühling *et al.* (16) showed that cyanophage infection played an important role in the succession of *Synechococcus* genotypes. The cyanomyovirus diversity results obtained from this study demonstrated that cyanophage infection can potentially control the dominant species of cyanobacteria in Kranji Reservoir.

It was previously unclear whether cyanophages responded to changes in *Synechococcus* assemblages or if phage infection controlled the *Synechococcus* assemblage? Throughout this study, we found that fluctuations in water quality affected the growth of the *Synechococcus* community (during August 2008 and September 2008) and, subsequently, the genotype of cyanophages. With the emergence of dominant genotypes, cyanophages could be an important factor controlling the diversity of *Synechococcus* populations by “killing the winner” (during October 2008 and November 2008). As the host community diversifies, this, in turn, leads to an increase in the diversity of cyanophages. Thus, cyanophage diversity could potentially be used as an indicator of a shift in the strains of particular species of cyanobacteria.

## Conclusion

In order to deepen our understanding of how cyanophages respond to changes in host population dynamics and vice versa, a comprehensive sampling to capture the development and collapse of relevant genotypes will be required. We suggest that instead of regular monthly sampling, sampling should be conducted intensively during three main periods of a bloom cycle, *i.e.* initial peaking of the bloom, during the bloom event, and the die-off period. This will enable a better understanding of the short-term dynamics of phage and host diversity.

## Supplementary Information



## Figures and Tables

**Table 1:** Environmental variables characterizing Stations A, B, and C from August 2008 to February 2009

Variables	Station A			Station B			Station C		
		
	Min. (month)	Max. (month)	Mean	Min. (month)	Max. (month)	Mean	Min. (month)	Max. (month)	Mean
Chlorophyll-a (μg L^−1^)	41.9 (Oct)	100.1 (Feb)	72.46	40.2 (Nov)	109.6 (Sept)	71.89	10.1 (Sept)	1584.8 (Nov)	301.39
Total nitrogen, TN (mg N L^−1^)	0.64 (Aug)	2.50 (Sept)	1.19	0.61 (Aug)	3.67 (Sept)	1.23	1.01 (Aug)	2.91 (Dec)	2.09
Total Phosphorus, TP (mg P L^−1^)	0.062 (Aug)	0.101 (Feb)	0.078	0.027 (Jan)	0.110 (Feb)	0.071	0.062 (Aug)	0.211 (Feb)	0.128
TN/TP	10.34 (Aug)	35.81 (Sept)	15.91	8.40 (Nov)	33.89 (Sept)	17.32	10.29 (Oct)	37.50 (Sept)	18.64
Calcium, Ca^2+^ (mg L^−1^)	16.88 (Dec)	22.77 (Oct)	18.76	17.89 (Dec)	21.94 (Oct)	19.43	15.56 (Nov)	24.41 (Aug)	21.2
Magnesium, Mg^2+^ (mg L^−1^)	1.88 (Nov)	2.77 (Feb)	2.36	1.98 (Nov)	2.76 (Feb)	2.41	1.04 (Nov)	3.65 (Aug)	2.56
Dissolved oxygen, DO (mg L^−1^)	4.48 (Oct)	7.65 (Jan)	6.07	4.09 (Nov)	7.12 (Sept)	5.83	2.97 (Aug)	8.68 (Oct)	5.85
Temperature (°C)	26.7 (Jan)	30 (Sept)	28.68	26.5 (Jan)	30.7 (Sept)	28.84	26.2 (Nov/Jan)	29.1 (Oct)	27.83
Conductivity (μs cm^−1^)	185.35 (Oct)	241.95 (Sept)	201.6	183.6 (Jan)	239.65 (Feb)	202.08	106.5 (Nov)	302.85 (Aug)	209.09
Turbidity (NTU)	25.05 (Aug)	51.6 (Sept)	35.55	13.25 (Nov)	86.5 (Sept)	32.66	21 (Aug)	224.5 (Nov)	75.49
pH	7.58 (Oct)	8.8 (Feb)	8.13	7.34 (Nov)	9.01 (Sept)	8.15	7.5 (Nov)	9.7 (Feb)	8.34
Total dissolved solid, TDS (g L^−1^)	0.103 (Nov)	0.134 (Feb)	0.114	0.104 (Nov)	0.134 (Feb)	0.114	0.063 (Nov)	0.184 (Aug)	0.120
Total suspended solid, TSS (mg L^−1^)	17 (Oct)	25.2 (Jan)	19.8	10.8 (Dec)	28 (Sept)	20.69	10.4 (Sept)	210 (Nov)	51.62
Secchi depth (cm)	43 (Aug)	67.5 (Oct)	52.29	39.5 (Aug)	69 (Dec)	57.21	19 (Nov)	70 (Aug)	43.36

**Table 2:** Spatial cluster distributions of cyanomyoviruses

Sampling point/Cluster	Number of clones (% of cluster from each station)						Total clones for each station

	β	α	γ	δ	ζ	ɛ	
Station A	17 (51.5)	2 (6.1)	1 (3.0)	3 (9.1)	4 (12.1)	6 (18.2)	33
Station B	12 (44.4)	1 (3.7)	0	3 (11.1)	3 (11.1)	7 (25.9)	27 (with one outcluster)
Station C	12 (46.2)	4 (15.4)	0	3 (11.5)	5 (19.2)	2 (7.7)	26

**Table 3:** Temporal cluster distribution of cyanomyoviruses

Cluster/Sampling month	Number of clones							Total clones for each cluster	% of each cluster

	Aug 08	Sept 08	Oct 08	Nov 08	Dec 08	Jan 09	Feb 09		
β	3	10	10	1	7	5	5	41	48.2
α	1	1	1	1	1	0	2	7	8.2
γ	0	0	0	1	0	0	0	1	1.2
δ	9	0	0	0	0	0	0	9	10.6
ζ	0	0	4	4	1	0	3	12	14.1
ɛ	0	2	0	1	2	6	4	15	17.7

Total Clones	13	13	15	9*	11	11	11		

* November 2008 samples with 8 clones distributed to 5 clusters and 1 outcluster.

**Table 4:** Cluster distribution of cyanomyoviruses with different monsoon seasons

Monsoon/Cluster	Number of clones (% of cluster from each monsoon)						Total clones for each monsoon

	β	α	γ	δ	ζ	ɛ	
Southwest (Aug 08 and Sept 08)	13 (50.0)	2 (7.7)	0	9 (34.6)	0	2 (7.7)	26
Inter (Oct 08 and Nov 08)	11 (45.8)	2 (8.3)	1 (4.2)	0	8 (33.3)	1 (4.2)	24
Northeast (Dec 08 to Feb 09)	17 (47.2)	3 (8.3)	0	0	4 (11.1)	12 (33.3)	36
