# Supplementary material for: Population Dynamics of Cyanomyovirus in a Tropical Eutrophic Reservoir
Source: Microbes Environ. 2014 Dec 27;30(1):12–20. doi: 10.1264/jsme2.ME14039 (PMC4356459; doi:10.1264/jsme2.ME14039)
Supplement: Supplementary file 1 [file 30_12_s1.pdf]

Table S1 Environmental variables characterizing Station A from August 2008 to February 2009

Parameter/ Date	8-Aug-08	23-Sep-08	21-Oct-08	20-Nov-08	19-Dec-08	16-Jan-09	19-Feb-09
Chlorophyll-a (µg/L)	77.67	98.87	41.94	69.21	66.48	52.95	100.11
Total Nitrogen, TN (mg N/L)	0.64	2.51	0.87	0.93	1.16	1.12	1.18
Total Phosphorus, TP (mg P/L)	0.06	0.07	0.07	0.09	0.07	0.09	0.10
TN/TP	10.34	35.81	13.12	10.56	17.29	12.54	11.68
Calcium, Ca^{2+} (mg/L)	18.00	19.67	22.77	17.08	16.88	18.47	18.44
Magnesium, Mg^{2+} (mg/L)	2.41	2.20	2.69	1.88	2.09	2.49	2.77
Dissolved Oxygen (mg/ L)	5.47	6.44	4.48	5.28	6.87	7.65	6.31
Temperature (°C)	29.40	30.00	28.95	28.70	27.95	26.70	29.10
Conductivity (µs/cm)	213.00	210.20	185.35	185.50	189.80	185.40	241.95
Turbidity (NTU)	25.05	51.60	28.40	27.85	37.65	37.00	41.35
pH	7.93	7.96	7.59	7.83	8.60	8.20	8.80
Total Dissolved Solid, TDS (g/L)	0.13	0.11	0.10	0.10	0.11	0.11	0.13
Total Suspended Solid, TSS (mg/L)	18.08	18.80	17.00	20.10	21.40	25.20	18.00
Secchi Depth (cm)	43	50	67.5	52	57	50	46.5
Salinity (ppt)	0.10	0.10	0.10	0.10	0.10	0.10	0.10

Table S2 Environmental variables characterizing Station B from August 2008 to February 2009

Parameter/ Date	8-Aug-08	23-Sep-08	21-Oct-08	20-Nov-08	19-Dec-08	16-Jan-09	19-Feb-09
Chlorophyll-a (µg/L)	96.75	109.56	77.03	46.18	51.93	51.08	70.77
Total Nitrogen, TN (mg N/L)	0.66	3.67	0.93	0.61	0.88	0.74	1.10
Total Phosphorus, TP (mg P/L)	0.06	0.11	0.07	0.07	0.06	0.03	0.11
TN/TP	11.85	33.90	14.13	8.40	15.62	27.41	9.93
Calcium, Ca^{2+} (mg/L)	18.97	19.54	21.94	18.41	17.90	20.93	18.36
Magnesium, Mg^{2+} (mg/L)	2.49	2.15	2.73	1.98	2.16	2.62	2.76
Dissolved Oxygen (mg/ L)	4.82	7.12	5.64	4.10	6.23	6.29	6.62
Temperature (°C)	29.80	30.70	29.50	28.70	27.95	26.50	28.75
Conductivity (µs/cm)	213.30	213.40	191.70	185.05	187.90	183.60	239.65
Turbidity (NTU)	29.80	86.50	21.40	13.25	24.40	25.75	27.50
pH	8.88	9.01	7.69	7.34	8.30	8.00	7.85
Total Dissolved Solid, TDS (g/L)	0.13	0.12	0.11	0.10	0.11	0.11	0.13
Total Suspended Solid, TSS (mg/L)	26.00	28.00	17.20	16.20	10.80	27.80	18.80
Secchi Depth (cm)	39.50	46.50	64.50	68.50	69.00	60.00	52.50
Salinity (ppt)	0.10	0.10	0.10	0.10	0.10	0.10	0.10

Table S3 Environmental variables characterizing Station C from August 2008 to February 2009

Parameter/ Date	8-Aug-08	23-Sep-08	21-Oct-08	20-Nov-08	19-Dec-08	16-Jan-09	19-Feb-09
Chlorophyll-a (µg/L)	12.84	10.14	110.83	1584.76	131.71	75.90	183.56
Total Nitrogen, TN (mg N/L)	1.01	2.85	1.79	2.36	2.91	1.33	2.43
Total Phosphorus, TP (mg P/L)	0.06	0.08	0.17	0.17	0.11	0.08	0.21
TN/TP	16.26	37.50	10.29	13.50	25.45	16.00	11.51
Calcium, Ca^{2+} (mg/L)	24.41	23.08	23.86	15.56	19.19	22.54	19.76
Magnesium, Mg^{2+} (mg/L)	3.65	2.25	2.61	1.04	2.35	3.11	2.93
Dissolved Oxygen (mg/ L)	2.97	4.27	8.68	4.10	6.95	6.37	7.60
Temperature (°C)	28.50	28.65	29.10	26.20	27.45	26.20	28.70
Conductivity (µs/cm)	302.85	217.15	181.15	106.50	196.55	195.95	263.50
Turbidity (NTU)	21.00	22.75	64.00	224.50	66.95	51.20	78.05
pH	7.98	7.66	7.97	7.50	9.15	8.40	9.70
Total Dissolved Solid, TDS (g/L)	0.18	0.12	0.10	0.06	0.11	0.11	0.15
Total Suspended Solid, TSS (mg/L)	19.25	10.40	26.00	210.00	30.20	30.80	34.67
Secchi Depth (cm)	70.00	60.00	45.00	19.00	39.50	40.00	30.00
Salinity (ppt)	0.10	0.10	0.10	0.10	0.10	0.10	0.10

Table S4 Comparison of closest relatives of the sequenced g20 (cyanomyoviruses) clones of Kranji Reservoir at the amino acid level. The clone names are labeled according to the format WXYZ, where W is sampling location, X is sampling date, Y is phage family (*Myoviridae*) and Z is the sample number.

Clone Name	Length (amino acid)	*Cluster	Closet Relatives to clone	Identity % (coverage)[a]	Source (Cluster)[b]
KRA0808M1	181	δ	MC3	95	Laurentian Great Lake
KRA0808M2	181	δ	g20_12_56_1%_NEQ	84	Atlantic Meridional Transect
KRA0808M3	181	δ	PFW-CF6	94	Floodwater of a Japanese paddy field at Aichi, Anjo (CSP/δ-PFW2)
KRA0808M4	183	β	MC38	85	Ballast sample, Burns harbor (C3)
KRA0808M5	181	β	d04	87	Lake Bourget, France
KRA0908M1/M5	181	β	KuCf-Apr13-7	87	Paddy field soil Japan: Aomori, Kuroishi (β- PFS II)
KRA0908M2	181	β	AnCf-Apr11-5	81	Paddy field soil Japan: Aichi, Anjo(β- PFS I)
KRA0908M3	183	β	OTU7	81 (98%)	Chesapeake Bay (N4)
KRA0908M4	181	β	KuCf-Apr13-7	86	Paddy field soil Japan: Aomori, Kuroishi (β- PFS II)
KRA1008M1/M2	183	β	MC38	81	Ballast sample, Burns harbor (C3)
KRA1008M3	181	β	KuCf-Apr13-7	84	Paddy field soil Japan: Aomori, Kuroishi (β- PFS II)
KRA1008M4	181	β	KuCf-Apr13-7	87	Paddy field soil Japan: Aomori, Kuroishi (β- PFS II)
KRA1008M5	181	β	KuCf-Apr13-	88	Paddy field soil Japan: Aomori,

/1108M1			7		Kuroishi (β- PFS II)
KRA1108M2	181	ζ	CUL02M-11	82 (99%)	Cultus Lake, BC, Canada (J)
KRA1108M3	181	γ	PFW-CM11	91	Floodwater of a Japanese paddy field at Aichi, Anjo (γ- PFW- VI)
KRA1108M4	181	α	PFW-NoF13	96	Floodwater of a Japanese paddy field at Aichi, Anjo (α- PFW-III)
KRA1108M5	181	ζ	CUL02M-11	82 (98%)	Cultus Lake, BC, Canada (J)
KRA1208M1	181	ζ	CUL02M-11	83 (98%)	Cultus Lake, BC, Canada (J)
KRA1208M2	183	β	MC38	81	Ballast sample, Burns harbor (C3)
KRA1208M3	181	ε	VC63_A8	85 (98%)	Sandusky Bay (C2)
KRA1208M4	181	β	PFW-CM33	86	Floodwater of a Japanese paddy field at Aichi, Anjo (β- PFW-V)
KRA0109M1	181	ε	VC63_A8	92	Sandusky Bay (C2)
KRA0109M3	181	ε	VC63_A8	92	Sandusky Bay (C2)
KRA0109M4	181	β	DC05 g20	93	Dianci lake, Yunnan China
KRA0109M5	181	ε	VC63_A8	92	Sandusky Bay (C2)
KRA0209M1	181	α	PFW-NoF13	96	Floodwater of a Japanese paddy field at Aichi, Anjo (α- PFW-III)
KRA0209M2 /M3	181	ε	o02	76	Lake Bourget, France
KRA0209M4	181	ζ	CUL02M-14	72 (99%)	Cultus Lake, BC, Canada (J)
KRA0209M5	182	β	MC27	95	Sandusky Bay (C3)
KRB0808M1	182	δ	SE5	96	Skidaway Estuarine Surface Water (I)
KRB0808M2 /M4	181	δ	MC4	94	Laurentian Great Lake

KRB0908M1	181	α	PFW-NoF13	96	Floodwater of a Japanese paddy field at Aichi, Anjo (α- PFW-III)
KRB0908M2	181	ε	VC63_A8	84 (98%)	Sandusky Bay (C2)
KRB0908M3	181	β	BES02A-4	83 (99%)	Beaufort Sean, Arctic Ocean (Cluster 6)
KRB1008M1	183	β	MC38b	81	Ballast sample, Burns harbor (C3)
KRB1008M2	183	β	MC38	81	Ballast sample, Burns harbor (C3)
KRB1008M3	183	β	OTU7	96 (98%)	Chesapeake Bay (N4)
KRB1008M4	183	β	MC38	81	Ballast sample, Burns harbor (C3)
KRB1008M5	181	ζ	VC63_F8	70	Sandusky Bay (C2)
KRB1108M1	183	ζ	PFW-CM14	59 (99%)	paddy floodwater
KRB1108M2	181	ε	VC63_A8	93	Sandusky Bay (C2)
KRB1108M3	181	ζ	VC63_F8	72	Sandusky Bay (C2)
KRB1108M4	181	-	SS4716	60 (99%)	DCM of Sargasso Sea
KRB1208M1	181	β	KuCf-Apr13-7	88	Paddy field soil Japan: Aomori, Kuroishi (β- PFS- II)
KRB1208M2	183	β	MC38	81	Ballast sample, Burns harbor (C3)
KRB1208M3	181	ε	VC63_A8	93	Sandusky Bay (C2)
KRB1208M4	181	β	DC05	94	Dianchi Lake, Yunnan China
KRB0109M1	181	ε	VC63_A8	85	Sandusky Bay (C2)
KRB0109M2	181	ε	VC63_A8	93	Sandusky Bay (C2)
KRB0109M3	181	β	DC05	93	Dianchi Lake, Yunnan China

KRB0209M1 /M5	182	β	MC27	95	Sandusky Bay (C3)
KRB0209M2	181	ε	o02	76	Lake Bourget, France
KRB0209M3	181	ε	VC63_A8	93	Sandusky Bay (C2)
KRB0209M4	181	β	VC63_B8	92	Sandusky Bay (C2)
KRC0808M1	182	α	MC10	96	Sandusky Bay Particulate (C3)
KRC0808M2	181	δ	MC4	94	Laurentian Great Lake
KRC0808M3	181	δ	g20_44_68_1%	85	Atlantic Meridional Transect
KRC0808M4	183	β	OTU7	81 (98%)	Chesapeake Bay (N4)
KRC0808M5	182	δ	PFW-NoF20	98	Floodwater of a Japanese paddy field at Aichi, Anjo (CSP/δ-PFW2)
KRC0908M1	181	ε	VC63_A8	83 (98%)	Sandusky Bay (C2)
KRC0908M2	181	β	MC27	82	Sandusky Bay (C3)
KRC0908M3	183	β	PFW-NoF21	80	Floodwater of a Japanese paddy field at Aichi, Anjo (β)
KRC0908M4	181	β	MC27	82	Sandusky Bay (C3)
KRC0908M5	181	β	KuCf-Apr13-7	85 (99%)	Paddy field soil Japan: Aomori, Kuroishi (β- PFS- II)
KRC1008M1	181	ζ	VC63_F8	65 (98%)	Sandusky Bay (C2)
KRC1008M2 /M4	181	ζ	VC63_F8	68 (98%)	Sandusky Bay (C2)
KRC1008M3	181	α	PFW-CF1	86	Floodwater of a Japanese paddy field at Aichi, Anjo (α-PFW-II)
KRC1008M5	181	β	KuCf-Apr13-7	83 (99%)	Paddy field soil Japan: Aomori, Kuroishi (β- PFS- II)

KRC1208M1	181	β	KuCf-Apr13-7	84 (99%)	Paddy field soil Japan: Aomori, Kuroishi (β- PFS- II)
KRC1208M2	181	α	PFW-NoF13	96	Floodwater of a Japanese paddy field at Aichi, Anjo (α- PFW-III)
KRC1208M3	184	β	PFW-NoF21	83 (95%)	Floodwater of a Japanese paddy field at Aichi, Anjo (β)
KRC0109M1	181	ε	VC63_A8	93	Sandusky Bay (C2)
KRC0109M2	181	β	DC05	93	Dianchi Lake, Yunnan China
KRC0109M3 /M4	183	β	OTU7	80	Chesapeake Bay (N4)
KRC0209M1	181	α	PFW-NoF13	97	Floodwater of a Japanese paddy field at Aichi, Anjo (α- PFW-III)
KRC0209M2	181	ζ	CUL02M-11	83	Cultus Lake, BC, Canada (J)
KRC0209M3	181	β	KuCf-Apr13-7	84	Paddy field soil Japan: Aomori, Kuroishi (β- PFS- II)
KRC0209M4	181	ζ	CUL02M-11	83	Cultus Lake, BC, Canada (J)

[*] Cluster assigned in this study

[a] Percentage of coverage between sequence of this study and previous study

[b] Cluster assigned by the originated study
